# Electrophysiological and Behavioral Responses of *Thrips hawaiiensis* (Thysanoptera: Thripidae) to the Floral Volatiles of the Horticultural Plant *Magnolia grandiflora* (Magnoliales: Magnoliaceae)

**DOI:** 10.3390/insects16060633

**Published:** 2025-06-16

**Authors:** Tao Zhang, Yuping Yang, Filippo Maggi, Feiyu Jiang, Rongrong Yuan, Lujie Huang, Xueyan Zhang, Yu Cao, Yulin Gao

**Affiliations:** 1 School of Pharmacy, Guizhou University of Traditional Chinese Medicine, Guiyang 550025, China; zhangtao3185@126.com; 2 Guizhou Provincial Key Laboratory for Rare Animal and Economic Insect of the Mountainous Region, Key Laboratory of Surveillance and Management of Invasive Alien Species in Guizhou Education Department, Guiyang University, Guiyang 550005, China; 13037865352@163.com (Y.Y.); 18585025745@163.com (F.J.); 18185682620@163.com (R.Y.); 15585097265@163.com (L.H.); 18722905420@163.com (X.Z.); 3 School of Pharmacy, Chemistry Interdisciplinary Project (ChIP) Research Center, University of Camerino, Via Madonna delle Carceri, 62032 Camerino, Italy; filippo.maggi@unicam.it; 4 State Key Laboratory for Biology of Plant Diseases and Insect Pests, Institute of Plant Protection, Chinese Academy of Agricultural Sciences, Beijing 100193, China

**Keywords:** flower-dwelling thrips, behavioral responses, volatile organic compounds, electroantennography, host plant preference

## Abstract

Volatile cues are important in the host detection and discrimination of phytophagous insects, allowing them to find suitable hosts. Here, the electroantennography (EAG) and behavioral responses of female *Thrips hawaiiensis* to the floral volatiles of different plants, *Magnolia grandiflora* L. (Magnoliales: Magnoliaceae), *Gerbera jamesonii* Bolus (Asterales: Asteraceae), *Lilium brownii* Baker (Liliales: Liliaceae), and *Rosa rugosa* Thunb. (Rosales: Rosaceae), were studied. Y-tube olfactometer bioassays revealed that *M. grandiflora* was the preferred host for *T. hawaiiensis*. Fifty-two compounds were identified from the volatile profile of *M. grandiflora* by gas chromatography–mass spectrometry analysis, of which *β*-elemen (15.39%), bicyclogermacren (11.99%), and (*E*)-*α*-bisabolene (6.05%) showed the highest relative contents. The EAG bioassays revealed that the antennae of *T. hawaiiensis* could perceive these tested volatile compounds at different concentrations. In six-arm olfactometer bioassays, *T. hawaiiensis* showed significant positive responses to *β*-elemen and (*E*)-*α*-bisabolene at various concentrations, and 10 μg/μL was found to be the most attractive concentration for *β*-elemen, and 50 and 100 μg/μL for (*E*)-*α*-bisabolene. Based on the four-arm olfactometer bioassays, *β*-elemen was more attractive than (*E*)-*α*-bisabolene to *T. hawaiiensis* when compared at their optimal concentrations. Therefore, *T. hawaiiensis* could perceive and distinguish the floral volatiles from the preferred host plant (*M. grandiflora*). These findings assist in better understanding the mechanism of host preferences of *T. hawaiiensis* from a chemoecological perspective. In particular, *β*-elemen showed the greatest potential to be developed as a novel attractant for the monitoring and control of *T. hawaiiensis*.

## Simple Summary

The aim is to understand the host preferences of *Thrips hawaiiensis* from a chemoecological perspective. The olfactory responses of female *T. hawaiiensis* to the floral volatiles of different plants were studied using electroantennography (EAG) and behavioral bioassays in different types of olfactometers. Then, the components of the volatile profile of the preferred host (*Magnolia grandiflora* flower) were further analyzed by gas chromatography–mass spectrometry. According to the EAG and behavioral bioassays, we found that *T. hawaiiensis* showed significant olfactory preference to *β*-elemen, bicyclogermacren, and (*E*)-*α*-bisabolene, which were three main components identified from the volatiles of *M. grandiflora* flowers. In short, our results indicated that semiochemical volatiles played important roles in searching the preferred host plant species of *T. hawaiiensis*. In addition, *β*-elemen showed the greatest potential to be used in the integrated management of *T. hawaiiensis*.

## 1. Introduction

Thrips are members of the order Thysanoptera, which includes more than 6000 described extant species. They are opportunistic and ubiquitous insects with small bodies, and many species are preadapted to an invasive lifestyle. The body length of the adult ranges from less than 1 mm to only a few millimeters, so they can be carried by the wind over long distances. *Thrips hawaiiensis* (Morgan, 1913) (Thysanoptera: Thripidae) is a common flower-thrips species that is native to the Asia and Pacific regions [1]. As a result of increasing international trade, it is now distributed in Asia, Australia, America, Africa, and Europe [2,3,4,5] and has become an important agricultural and horticultural pest worldwide.

*Thrips hawaiiensis* has a wide host range among crop plants, including various ornamental plants, fruits, and vegetables [6,7,8,9]. However, *T. hawaiiensis* shows clear host preferences in the field, with different population sizes and associated degrees of damage among different host plant species, e.g., *Gardenia jasminoides* (Gentianales: Rubiaceae), *Hydrangea macrophylla* L. (Saxifragales: Saxifragaceae), and 19 other plants [8,9]. Particularly, when *T. hawaiiensis*, *Thrips flavidulus* Bagnall (Thysanoptera: Thripidae), *Frankliniella occidentalis* Pergande (Thysanoptera: Thripidae), and *Thrips coloratus* Schmutz (Thysanoptera: Thripidae) coexisted on *M. grandiflora* flowers, *T. hawaiiensis* showed a significantly higher number than the other three thrips species, indicating the dominant position of this thrips on this host plant [10]. In addition, *T. hawaiiensis* prefers to feed and live in flowers, rather than in other plant structures [1,6,11]. Therefore, this thrip pest not only shows host preferences, but also preferences for different parts of the host plant.

There is increasing evidence that olfactory cues are important for host-searching by flower-inhabiting thrips, including *T. hawaiiensis*, and that thrips use plant volatiles to locate more suitable host plants, as it has been reported that *F. occidentalis* were attracted to the volatiles of *Rosa rugosa* (Rosales: Rosaceae) flower and *T. hawaiiensis* were attracted to the volatiles of *G. jasminoides* flower [12,13,14]. These findings would be useful in building a push–pull system for thrips pest control, thereby reducing the need for chemical sprays [15,16].

According to our previous studies, *Magnolia grandiflora* L., *Gerbera jamesonii* Bolus, *Lilium brownii* Baker, and *R. rugosa* Thunb. are four host plants of *T. hawaiiensis*, and the population fitness of *T. hawaiiensis* differs among these hosts, indicating this pest could cause different degrees of damage to different plant species [9,10,14]. In the present study, the olfactory responses of *T. hawaiiensis* to these different plant flowers above were studied using electroantennography (EAG) and behavioral bioassays in Y-tube, six-arm, and four-arm olfactometers. Our study provides new information for further exploring the mechanism of host selection and damage in *T. hawaiiensis*, based on the chemoecology of its interaction with potential host plants. The findings of this study also provide new information that will be useful for ecological regulation or control of thrips pests. In particular, these results could provide candidate compounds for the development of new attractants/repellents for the integrated management of *T. hawaiiensis*.

## 2. Materials and Methods

### 2.1. Insects and Host Plants

*Thrips hawaiiensis* adults were collected from different weeds, as well as vegetable and flowering plant species, in the Nanming district (106°77′78.24″ E, 26°55′73.56″ N), Guiyang area, Guizhou Province, China. After being taken back to the laboratory, these thrips were reared on green bean pods of *Phaseolus vulgaris* L. (Fabales: Fabaceae) in plastic containers (20 cm × 14 cm × 9 cm) with snap-on lids and used to establish laboratory colonies [17]. The thrips colonies were reared for more than five generations before being used for bioassays and were kept in a climate-controlled room (RTOP-400Y, Tuopu Yunnong Technology Co., Ltd., Hangzhou, China) at 25 °C ± 1 °C, 70% ± 5% relative humidity under a 14 h light–10 h dark photoperiod.

Different flower plant species, *M. grandiflora*, *G. jamesonii*, *L. brownii*, and *R. rugosa*, were grown in greenhouses in the nursery of Guiyang University, Guizhou Province, China [8]. Greenhouses were kept free of pests by insect-proof netting, and no insecticides were applied to these plants. Plant flowers at anthesis were collected for olfactory tests and gas chromatography–mass spectrometry (GC-MS) analysis.

### 2.2. Y-Tube Olfactometer Bioassays

The olfactory responses of *T. hawaiiensis* to the volatiles of different plant flowers were tested in a Y-tube olfactometer by the method of Colazza et al. [18] and Cao et al. [17]. Here, we made two types of comparisons: (1) plant flowers (each 20.0 g) versus clean air (CA); and (2) all the possible pairings of these four plant flowers (20.0 g each). Because thrips females are more sensitive to volatiles from host plants [14], only *T. hawaiiensis* females were used in this study. For each comparison, 60 *T. hawaiiensis* females (2–3 days old) were tested individually, and flower material was replaced with an equal quantity after testing 10 individuals. Before each test treatment, *T. hawaiiensis* adults were initially starved for 6 h. Airflow was set at 200 mL/min. All bioassays were done between 09:00 and 17:00 at room temperature (25 °C ± 2 °C).

### 2.3. GC-MS Analysis

According to the Y-tube olfactometer bioassays, as *T. hawaiiensis* were most attracted to the mixture of volatile organic compounds (VOCs) from *M. grandiflora* flowers, the components of *M. grandiflora* floral VOCs were further analyzed. The collected VOCs were analyzed by solid phase microextraction–gas chromatography–mass spectrometry (SPME–GC–MS) (HP6890/5975C, Agilent Technologies, Santa Clara, CA, USA), as detailed in Báez et al. [19] and Cao et al. [13]. The chemical identities of the main peaks in the chromatograms were determined by comparing the mass spectra of compounds with those in databases (NIST 2017 and WILEY 275) [20].

### 2.4. Behavioral Responses of T. hawaiiensis to Main M. grandiflora VOCs

*β*-Elemen, bicyclogermacren, and (*E*)-*α*-bisabolene were the most abundant compounds that were identified from the VOCs profile of *M. grandiflora* (the most attractive plant species). Therefore, the behavioral responses of *T. hawaiiensis* to these compounds at different concentrations (0.1, 1, 10, 50, and 100 μg/μL) were further tested in different types of olfactometers.

### 2.5. Odor Stimuli

Solutions of *β*-elemen (MedChemExpress, Monmouth Junction, NJ, USA), bicyclogermacren (Sigma-Aldrich, St. Louis, MO, USA), and (*E*)-*α*-bisabolene (Sigma-Aldrich) in mineral oil (Sigma-Aldrich) were prepared at different concentrations for EAG and olfactometer bioassays and were stored at −20 °C until use.

### 2.6. Electroantennography Tests

In brief, a data acquisition collector, a stimulus flow controller, an AC/DC amplifier, a single-ended probe, and a micromanipulator were the five main components of the EAG system (SYNTECH, IDAC-2, Kirchzarten, Germany). The antennal sensitivity of *T. hawaiiensis* females (2–3 days old) to increasing concentrations of the three test compounds was evaluated by EAG using a technique described elsewhere [21,22,23]. To achieve better contact with the electrodes, the antennae of *T. hawaiiensis* females were excised at the groove between antennal segments 6 and 7 (away from the head). Then, the recording electrode was placed in contact with the last antennal segment of the thrips, and the neutral electrode was inserted into the base of the head. Mineral oil (10 μL) was used as a control; each compound was separately diluted in mineral oil to obtain different concentrations; and 10 μL of each compound at each concentration (0.001, 0.01, 0.1, 1, and 10 μg/μL) was used as the stimulus. Each compound at each dose was adsorbed onto a filter paper strip inserted in a Pasteur pipette, which was used as an odor cartridge. The measurements were conducted as described by Cao et al. [23].

### 2.7. Six-Arm Olfactometer Bioassays

The behavioral responses of *T. hawaiiensis* females to different doses of each of the three compounds [*β*-elemen, bicyclogermacren, and (*E*)-*α*-bisabolene] were also evaluated in a six-arm olfactometer using the method of Turlings et al. [24] with necessary modifications. The six-arm olfactometer consisted of a central chamber (120 mm internal diameter) with six arms (60 mm length and 15 mm internal diameter), and details were described in our previous study [25]. Solutions of these three compounds (0.1, 1, 10, 50, and 100 μg/μL, respectively) were also prepared with mineral oil, which were adsorbed onto a filter paper disk (1.0 cm diameter) and were used as stimuli (10 μL of each compound solution). Mineral oil (10 μL) was used as the control. In the six-arm olfactometer, each odor source was driven to thrips by the airflow at a flow rate of 200 mL min^−1^. Unmated female *T. hawaiiensis* adults (2–3 days old) were starved for 6 h and were then introduced into the olfactometer in groups of 200 individuals. The numbers of *T. hawaiiensis* that entered the arms of the olfactometer were counted and considered to have made a choice for an odor source, which should be accomplished within 30 min. Bioassays were repeated five times and were conducted between 09:00 and 17:00 at room temperature (25 °C ± 2 °C).

### 2.8. Four-Arm Olfactometer Bioassays

As tested in the six-arm olfactometer, bicyclogermacren was not attractive to *T. hawaiiensis* at any of the tested concentrations. The most attractive concentration of *β*-elemen was 10 μg/μL, and those of (*E*)-*α*-bisabolene were 50 and 100 μg/μL. Hence, the attractiveness of *β*-elemen and (*E*)-*α*-bisabolene at their optimal concentrations was compared in further four-arm bioassays [23,26], which only differed in the number of olfactometer arms as compared with the six-arm olfactometer bioassay described in the preceding section. The airflow was also maintained at 200 mL min^−1^. Female *T. hawaiiensis* adults were introduced in groups of 180 individuals, with five replicates.

### 2.9. Statistical Analyses

The null hypothesis that *T. hawaiiensis* adults showed no preference for either Y-tube arm (a response equal to 50:50) was analyzed using a chi-square goodness-of-fit test (in all cases, df = 1). The numbers of thrips showing preferences for odors in the 6-arm and 4-arm olfactometers were subjected to analysis of variance (ANOVA), followed by Tukey’s honestly significant difference (HSD) test (*p* < 0.05) for separation of means. All statistical analyses were performed using SPSS 18.0 for Windows (SPSS Inc., Chicago, IL, USA).

## 3. Results

### 3.1. Y-Tube Olfactometer Bioassays

**Figure 1:**
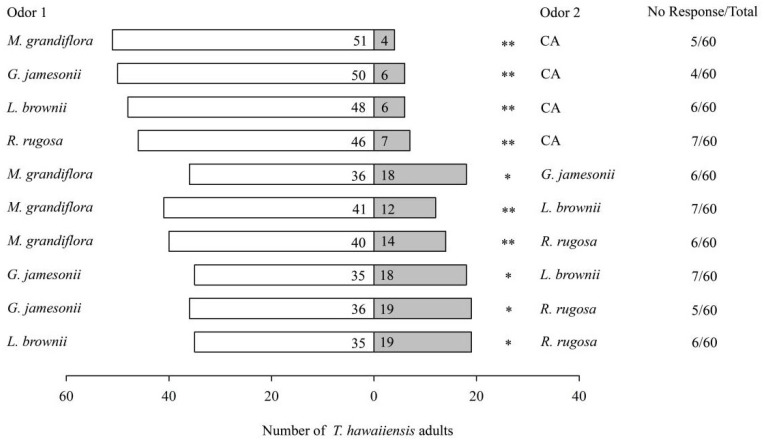
Olfactory responses of female *T. hawaiiensis* to different plant flowers. CA: clean air. Asterisks indicate highly significant (** *p* < 0.01) and significant (* *p* < 0.05) differences in selectivity of *T. hawaiiensis* between two odors by *χ*^2^ test.

When provided with different floral volatiles versus clean air (CA), female *T. hawaiiensis* exhibited significant preferences for *M. grandiflora* (*χ*^2^ = 40.16, *p* < 0.001), *G. jamesonii* (*χ*^2^ = 34.57, *p* < 0.001), *L. brownii* (*χ*^2^ = 32.67, *p* < 0.001), and *R. rugosa* (*χ*^2^ = 28.70, *p* < 0.001) over CA (Figure 1). When presented with pairs of floral volatiles of these four plant species, *T. hawaiiensis* females significantly preferred *M. grandiflora* to *G. jamesonii* (*χ*^2^ = 6.00, *p* = 0.014), *M. grandiflora to L. brownii* (*χ*^2^ = 15.87, *p* < 0.001), *M. grandiflora to R. rugosa*: *χ*^2^ = 12.52, *p* < 0.001), *G. jamesonii* to *L. brownii* (*χ*^2^ = 5.45, *p* = 0.02), *G. iamesonii* to *R. rugosa* (*χ*^2^ = 5.26, *p* = 0.022), and *L. brownii* to *R. rugosa* (*χ*^2^ = 4.74, *p* = 0.029) (Figure 1).

### 3.2. Analysis of M. grandiflora Flower Volatiles

Fifty-two components were identified in the floral VOCs profile of *M. grandiflora* (Table 1). *β*-Elemen showed the highest relative content (15.39%), followed by bicyclogermacren (11.99%), and (*E*)-*α*-bisabolene (6.05%). No other component showed a relative content exceeding 5% of the *M. grandiflora* flower volatiles, except for germacrene D (5.13%). Notably, *β*-elemen and bicyclogermacren are isomers of each other. There were 25 isomers of spathulenol in total, together accounting for 64.71% of the total VOCs profile. In addition, four components were not identified, with relative contents of 2.25%, 2.13%, 1.86%, and 1.05%, respectively.

### 3.3. Electroantennogram (EAG) Tests

**Figure 2:**
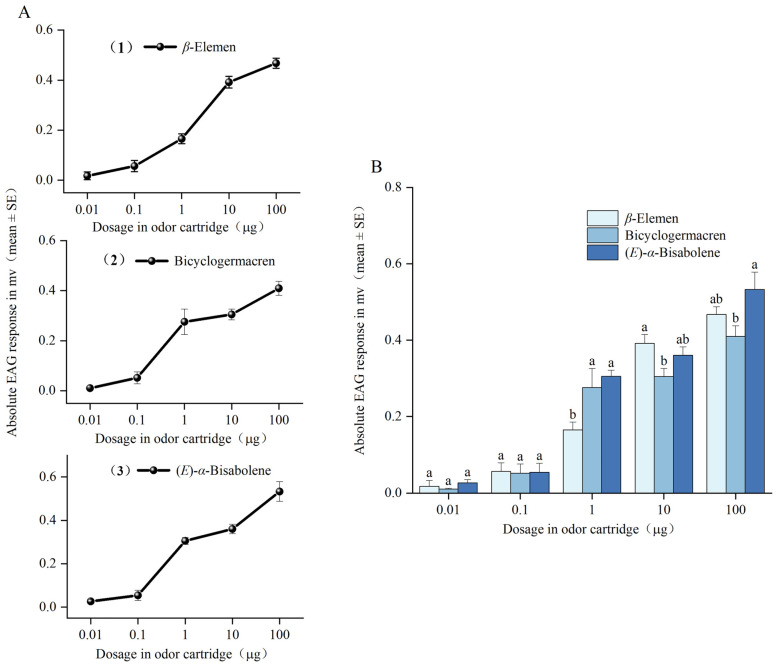
Electroantennography responses of female *T. hawaiiensis.* (**A**) EAG dose–response curves and (**B**) EAG responses of *T. hawaiiensis* to different doses of *β*-elemen, bicyclogermacren, and (*E*)-*α*-bisabolene. Mean values are shown. Different letters indicate significant differences among different compounds at the same dose (one-way analysis of variance followed by Tukey’s HSD test, *p* < 0.05).

The EAG responses of female *T. hawaiiensis* to increasing doses of *β*-elemen, bicyclogermacren, and (*E*)-*α*-bisabolene are shown in Figure 2A. In the dose range tested (starting from the 0.01 μg dose), all three compounds elicited typical sigmoid-shaped dose–responses in *T. hawaiiensis.* As determined by ANOVA, the thrips showed significantly different responses to the three compounds at the 1 μg dose (*F* = 96.69; df = 4, 20; *p* < 0.001), 10 μg (*F* = 33.64; df = 4, 20; *p* < 0.001), and 100 μg (*F* = 66.83; df = 4, 20; *p* < 0.001) (Figure 2B). Comparing the compounds at the 1 μg dose, the mean EAG values for bicyclogermacren and (*E*)-*α*-bisabolene were not significantly different from each other, but both were significantly higher than that for *β*-elemen. Comparing the compounds at the 10 μg dose, the mean EAG values were not significantly different between *β*-elemen and (*E*)-*α*-bisabolene, nor between bicyclogermacren and (*E*)-*α*-bisabolene, but were significantly higher for *β*-elemen than for bicyclogermacren. Comparing the compounds at the 100 μg dose, the mean EAG values were not significantly different between *β*-elemen and (*E*)-*α*-bisabolene, nor between *β*-elemen and bicyclogermacren, but were significantly higher for (*E*)-*α*-bisabolene than for bicyclogermacren.

### 3.4. Six-Arm Olfactometer Bioassays

**Figure 3:**
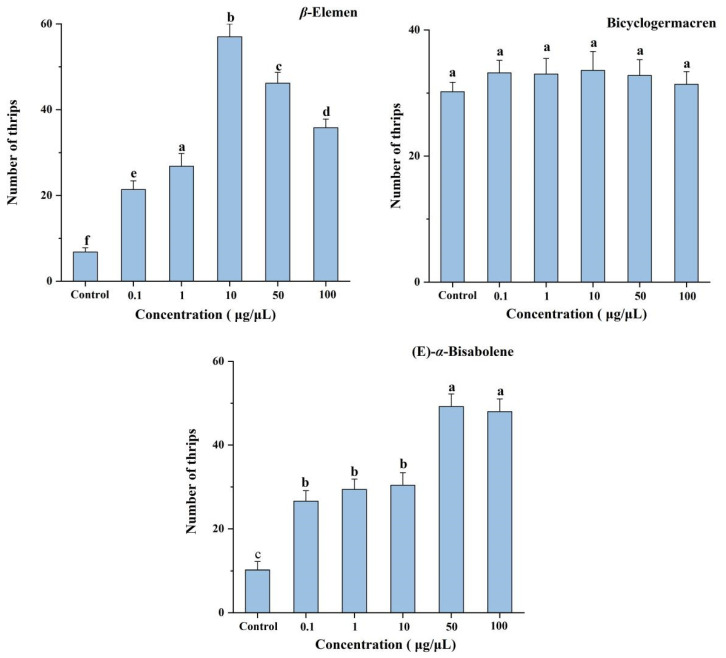
Olfactory responses of female *T. hawaiiensis* to *β*-elemen, bicyclogermacren, and (*E*)-*α*-bisabolene at different concentrations in a six-arm olfactometer. Control was mineral oil. Data are means ± SE. Different letters above bars indicate significant differences (one-way analysis of variance followed by Tukey’s HSD test, *p* < 0.05).

Six-arm bioassays showed that each concentration (0.1, 1, 10, 50, and 100 μg/μL) of *β*-elemen (*F* = 351.06; df = 5, 24; *p* < 0.001) and (*E*)-*α*-bisabolene (*F* = 176.04; df = 5, 24; *p* < 0.001) were significantly more attractive to *T. hawaiiensis*, compared with the control of mineral oil (Figure 3). The most attractive concentration of *β*-elemen was 10 μg/μL. For (*E*)-*α*-bisabolene, the most attractive concentrations were 50 and 100 μg/μL, but the degree of attractiveness did not differ between these two concentrations. However, bicyclogermacren at any concentration was not more attractive than mineral oil to *T. hawaiiensis* (*F* = 1.68; df = 5, 24; *p* = 0.18).

### 3.5. Four-Arm Olfactometer Bioassays

**Figure 4:**
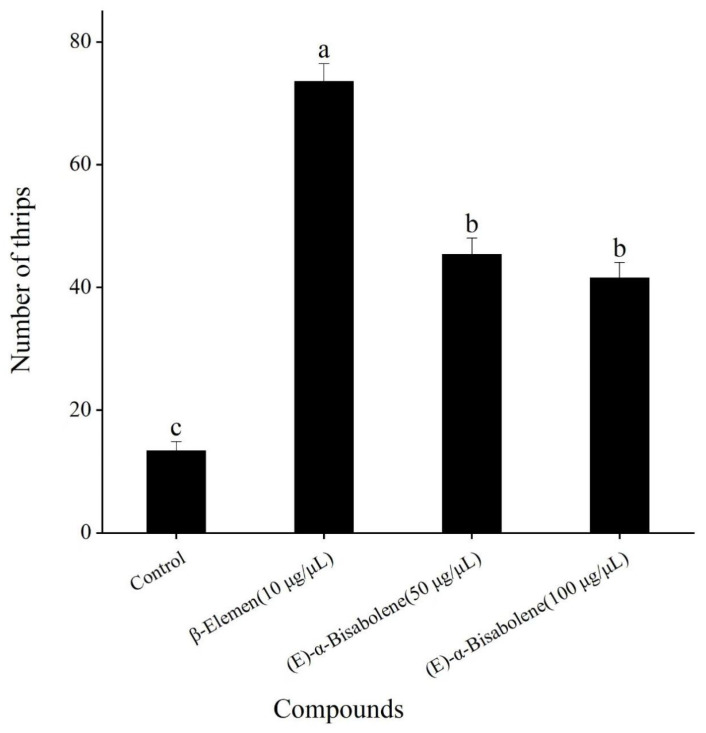
Olfactory responses of female *T. hawaiiensis* to *β*-elemen and (*E*)-*α*-bisabolene at their most attractive concentrations in a four-arm olfactometer. Control was mineral oil. Data are means ± standard errors (SE). Different lowercase letters on bars indicate significant differences (one-way analysis of variance followed by Tukey’s HSD test, *p* < 0.05).

As mentioned above, the most attractive concentration of *β*-elemen was 10 μg/μL, and the most attractive concentrations of (*E*)-*α*-bisabolene were 50 and 100 μg/μL. Compared with the control (mineral oil), although both *β*-elemen and (*E*)-*α*-bisabolene at their respective optimal concentrations were significantly more attractive to *T. hawaiiensis* (*F* = 284.03; df = 3, 16; *p* < 0.001) (Figure 4), *T. hawaiiensis* significantly preferred *β*-elemen to (*E*)-*α*-bisabolene, but showed no significant difference in the olfactory preference between 50 and 100 μg/μL of (*E*)-*α*-bisabolene.

## 4. Discussion

*Magnolia grandiflora* is recognized as a preferred host plant for *T. hawaiiensis*, and large numbers of this thrips species attack this species in the field [10]. Here, Y-tube olfactometer bioassays showed that *T. hawaiiensis* had clear olfactory preferences among the floral volatiles of four plant species, ranked as follows: *M. grandiflora* > *G. jamesonii* > *L. brownii* > *R. rugosa*. This rank order is consistent with the host plant fitness levels for *T. hawaiiensis*, as determined in our previous studies [9,10]. Similar results were reported for *Frankliniella occidentalis* (Thysanoptera: Thripidae), whose olfactory preferences were also closely related to their fitness levels among different host plants [13]. These results confirmed that volatile cues play a vital role in guiding insects as they search for suitable food, oviposition sites, or nutrient sources [27,28]. Our results may also partly explain why *T. hawaiiensis* populations reach different sizes and cause different degrees of damage to these four flowering plants.

The SPME–GC–MS analysis detected 52 compounds in the floral VOCs profile of *M. grandiflora*, among which *β*-elemen, bicyclogermacren, and (*E*)-*α*-bisabolene were the most abundant. These three main compounds were not detected in the floral VOCs profiles of *G. jamesonii*, *L. brownii*, or *R. rugosa* [13,14], which may explain why *T. hawaiiensis* prefers the floral VOCs of *M. grandiflora*. The EAG analyses revealed that these three main tested compounds were perceived by the peripheral olfactory system of *T. hawaiiensis* at a wide range of concentrations, and then their biological activity was further investigated in six- and four-arm olfactometers. The six-arm olfactometer bioassays indicated that *T. hawaiiensis* was significantly attracted to *β*-elemen and (*E*)-*α*-bisabolene at a range of concentrations, but not bicyclogermacren at any concentration. In addition, the most attractive concentrations were 10 μg/μL for *β*-elemen and 50 and 100 μg/μL for (*E*)-*α*-bisabolene. Furthermore, in the four-arm olfactometer bioassays, *T. hawaiiensis* preferred *β*-elemen to (*E*)-*α*-bisabolene when the two compounds were compared at their most attractive concentrations. Therefore, *β*-elemen has the greatest potential to be developed as a lure for *T. hawaiiensis*. Probably because of the differences in the cultivation conditions or geographical environment, the abundant compounds from the VOCs profile of *M. grandiflora* flowers differed in other research, while *β*-elemen was also one of the main compounds [19]. In addition, *β*-elemen is attractive to *Araecerus fasciculatus* (Coleoptera: Anthribidae) [29]. Thus, *β*-elemen has potential uses in the management of both field pests and insect pests of stored products. However, these results were obtained exclusively under laboratory conditions. Further investigation into field trapping tests is warranted to ensure the practical applicability of pest monitoring and control strategies.

Previous studies have reported that methyl anthranilate, linalool, (*E*-3,*E*-7)-4,8,12-trimethyltrideca-1,3,7,11-tetraene, (*Z*)-3-hexenyl tiglate, o-anisidine, and other volatile compounds are also attractive to *T. hawaiiensis* [14,30,31]. Therefore, the synergistic attractiveness of these volatile compounds as well as *β*-elemen to *T. hawaiiensis* should be further studied, with a range of tests at appropriate ratios and concentrations [32,33]. This would be useful to develop more efficient attractants for the control or integrated management of *T. hawaiiensis*. In addition, to our knowledge, only a small number of volatile compounds out of all of those emitted by host plants are involved in host detection by phytophagous insects [34,35,36]. Here, only the attractiveness of the most abundant components of the floral VOCs to thrips was assessed. The biological activity of minute amounts of other components (among the floral VOCs of *M. grandiflora*) for attracting *T. hawaiiensis* should be further investigated.

Although floral VOCs of *M. grandiflora* were able to elicit significant EAG and behavioral responses in female *T. hawaiiensis*, it is still unknown how *T. hawaiiensis* perceives these volatile stimuli by the olfactory neurons inside the antennal sensilla. Further studies should explore the molecular and cellular olfaction mechanism of host plant recognition in thrips [37,38,39]. This will help us to better understand the interaction between host plants and thrips at the chemoecological level and provide new information to elucidate the mechanism of the host preferences of thrips.

Our results suggest that, amongst the volatiles tested in this study, *β*-elemen showed the greatest potential to be developed as a new lure for *T. hawaiiensis*. Including this compound in attractants may make them more efficient and effective for the monitoring and control of this pest, especially when combined with sex pheromones and thrips parasitoid attraction [30,40].

## Figures and Tables

**Table 1:** Components of *M. grandiflora* flower volatiles.

Number	Compound	RI	Molecular Formula	Molecular Weight	Relative Peak Area (%)
1	*α*-Pinene	937	C_10_H_16_	136	1.17
2	Camphene	952	C_10_H_16_	136	0.11
3	Butanoic acid, 2-methyl-, 1-methylpropyl ester	971	C_9_H_18_O_2_	158	0.23
4	*β*-Pinene	979	C_10_H_16_	136	4.22
5	Myrcene	991	C_10_H_16_	136	1.02
6	Limonene	1031	C_10_H_16_	136	0.77
7	1,8-Cineole	1032	C_10_H_18_O	154	0.28
8	Terpinolene	1088	C_10_H_16_	136	0.13
9	Linalool	1099	C_10_H_18_O	154	0.23
10	Pinocarvone	1164	C_10_H_14_O	150	0.69
11	*α*-Terpineol	1189	C_10_H_18_O	154	0.15
12	Myrtenol	1213	C_10_H_16_O	152	0.17
13	Citronellol	1228	C_10_H_20_O	156	0.26
14	Geraniol	1255	C_10_H_18_O	154	0.17
15	*cis*-Myrtanol	1261	C_10_H_18_O	154	0.17
16	*trans*-Pinocarvyl acetate	1297	C_12_H_18_O_2_	194	0.41
17	*α*-Cubebene	1351	C_15_H_24_	204	0.22
18	*α*-Ylangene	1372	C_15_H_24_	204	0.19
19	*α*-Copaene	1376	C_15_H_24_	204	0.28
20	*β*-Cubebene	1390	C_15_H_24_	204	0.34
21	*β*-Elemene	1391	C_15_H_24_	204	15.39
22	Isocaryophyllene	1406	C_15_H_24_	204	0.31
23	*α*-Gurjunene	1409	C_15_H_24_	204	0.17
24	(*E*)-Caryophyllene	1419	C_15_H_24_	204	2.88
25	*β*-Copaene	1432	C_15_H_24_	204	0.36
26	*γ*-Elemene	1433	C_15_H_24_	204	1.62
27	Aromandendrene	1440	C_15_H_24_	204	1.06
28	Selina-5,11-diene	1447	C_15_H_24_	204	0.36
29	*α*-Humulene	1454	C_15_H_24_	204	1.01
30	Valerena-4,7(11)-diene	1460	C_15_H_24_	204	2.78
31	*cis*-Muurola-4(15),5-diene	1463	C_15_H_24_	204	0.69
32	*γ*-Gurjunene	1473	C_15_H_24_	204	1.09
33	*γ*-Muurolene	1477	C_15_H_24_	204	3.01
34	Germacrene D	1481	C_15_H_24_	204	5.13
35	*β*-Selinene	1486	C_15_H_24_	204	0.61
36	Bicyclogermacren	1495	C_15_H_24_	204	11.99
37	*δ*-Guaiene	1505	C_15_H_24_	204	4.47
38	(*E*)-*α*-Bisabolene	1512	C_15_H_24_	204	6.04
39	*γ*-Cadinene	1513	C_15_H_24_	204	0.68
40	*δ*-Cadinene	1524	C_15_H_24_	204	2.43
41	Germacrene B	1557	C_15_H_24_	204	0.39
42	(*E*)-Nerolidol	1564	C_15_H_24_	204	1.21
43	Spathulenol	1576	C_15_H_24_O	220	0.78
44	Caryophyllene oxide	1581	C_15_H_24_O	220	0.21
45	Globulol	1591	C_15_H_26_O	222	0.19
46	Isospathulenol	1638	C_15_H_24_O	220	0.37
47	*α*-Cadinol	1640	C_15_H_26_O	222	0.31
48	*n*-Nonadecane	1900	C_19_H_40_	268	0.37
49	*n*-Heneicosane	2100	C_21_H_44_	296	0.46
50	*n*-Tricosane	2300	C_23_H_48_	324	0.18
51	Cyclohexane,2-ethenyl-1,1-dimethyl-3-methylene-	2368	C_11_H_18_	150	3.77
52	Longiverbenone	2453	C_15_H_22_O	218	2.51
53	unidentified				2.25
54	unidentified				2.13
55	unidentified				1.86
56	unidentified				1.03

## Data Availability

The original contributions presented in this study are included in the article. Further inquiries can be directed to the corresponding author.
